# Specific patterns of genetic diversity among aromatic rice varieties in Myanmar

**DOI:** 10.1186/1939-8433-5-20

**Published:** 2012-08-01

**Authors:** Khin Myo Myint, Brigitte Courtois, Ange-Marie Risterucci, Julien Frouin, Khin Soe, Khin Maung Thet, Apichart Vanavichit, Jean-Christophe Glaszmann

**Affiliations:** 1 Plant Biotechnology Center, Myanma Agriculture Service, Yangon, Myanmar; 2 Cirad, UMR AGAP, Avenue Agropolis, 34398 Montpellier, France; 3 Department of Agricultural research, Yezin, Nay Pyi Taw, Myanmar; 4 Rice Science Center and Rice Gene Discovery, Kasetsart University, Kamphaeng Saen, Nakhon Pathom 73140 Thailand

**Keywords:** Oryza sativa, SSR, Genetic diversity, Aroma, *BADH2*

## Abstract

**Background:**

After observing peculiar rice varieties in Myanmar, in terms of classification in varietal groups and of grain quality, we focused on Myanmar varieties and analyzed variations at 19 microsatellite loci as well as sequences of the aroma gene *BADH2*.

**Results:**

Microsatellites were able to retrieve the well-established classification into Indica (isozyme group 1), Japonica (group 6, comprising temperate and tropical forms) and specific groups from the Himalayan foothills including some Aus varieties (group 2) and some aromatic varieties (group 5). They revealed a new cluster of accessions close to, but distinct from, non-Myanmar varieties in group 5. With reference to earlier terminology, we propose to distinguish a group “5A” including group 5 varieties from the Indian subcontinent (South and West Asia) and a group “5B” including most group 5 varieties from Myanmar. In Myanmar varieties, aroma was distributed in group 1 (Indica) and in group 5B. New *BADH2* variants were found. Some accessions carried a 43 bp deletion in the 3’ UTR that was not completely associated with aroma. Other accessions, all of group 5B, displayed a particular *BADH2* allele with a 3 bp insertion and 100% association with aroma.

**Conclusion:**

With the new group and the new alleles found in Myanmar varieties, our study shows that the Himalayan foothills contain series of non-Indica and non-Japonica varietal types with novel variations for useful traits.

**Electronic supplementary material:**

The online version of this article (doi:10.1186/1939-8433-5-20) contains supplementary material, which is available to authorized users.

## Background

Rice is the main cereal consumed by humans. *Oryza sativa,* the main rice species grown in the world, grows in a very wide range of ecological conditions all over the world and encompasses an extremely wide diversity; more than 106,800 accessions are stored in the International Rice Genebank in the Philippines (de Guzman, IRRI, personal communication).

The organization of *O. sativa* diversity has been of major interest for rice scientists since the early 20^th^ century, beginning with the pioneering work of ([Kato et al. 1928]), ([Matsuo 1952]), and ([Oka 1958]). The development of genetic markers, first enzymatic then DNA-based, enabled further refinement of the original classification ([Glaszmann 1987]; [Garris et al. 2005]). According to generally accepted knowledge, *O. sativa* accessions can be classified in two main groups, the indica group (isozyme group 1), which includes most tropical irrigated and rainfed lowland varieties, and the japonica group (isozyme group 6), a group with a wider range of agro-ecological adaptations. From a genetic viewpoint, the japonica group can be divided into a temperate component comprising irrigated varieties, and a tropical component comprising upland and high elevation varieties, although morphological studies showed that the two components represented a continuum ([Glaszmann and Arraudeau 1986]). In addition to these two major groups, two smaller groups were identified: the aus/boro group (isozyme group 2) comprising varieties from South Asia, and the sadri/basmati group (isozyme group 5) comprising irrigated varieties from all along the Himalayan border from Iran to Myanmar, including the world famous basmati rices from India and Pakistan and sadri from Iran ([Glaszmann 1987]). Additional groups such as deepwater rices from India and Bangladesh (enzymatic group 3) and rayada floating rices from Bangladesh (isozyme group 4) were identified in isozyme studies ([Glaszmann 1987]) but not confirmed with other markers. One possible reason for this discrepancy could be their under-representation in the samples analyzed, which in turn, may be explained by the small number of such accessions and the difficulties involved in obtaining seeds of these highly photoperiodic varieties.

Wide phenotypic diversity accompanies genetic diversity for all types of traits including grain characteristics. Rice is consumed mostly as cooked grain with little processing, and grain appearance, cooking quality, and taste are the factors that determine grain quality. Aromatic varieties are particularly appreciated by consumers in some countries and, for this reason, fetch a much higher price than non-aromatic varieties on these markets ([Unnevher et al. 1992]; [Singh et al. 2000]; [Juliano 2007]). Aroma in rice grain results from the production of many biochemical compounds ([Petrov et al. 1996]) of which the most important is 2-acetyl-1- pyrroline ([Buttery et al. 1982]). The presence of this molecule is genetically determined, but the intensity of this volatile compound is very much affected by the conditions of grain maturation and storage ([Singh et al. 2000]; [Fitzgerald et al. 2008]; [Gay et al. 2010]). Although the chemical pathway leading to the production of acetyl-pyrroline is not yet fully understood, a recessive gene annotated as a betaine aldehyde dehydrogenase (*BADH2*) located on chromosome 8 has been shown to play a key role in its synthesis ([Bradbury et al. 2005], [Bradbury et al. 2008], [Vanavichit et al. 2008]). Non-functional alleles of *BADH2* result in the accumulation of acetyl-pyrroline ([Chen et al. 2008];[Niu et al. 2008]). The diversity of this gene has been studied in a large collection of varieties and results showed that an 8 bp deletion in the seventh exon of *BADH2* causing a reading frame shift was present in most aromatic accessions, but other less frequent mutations associated with aroma were also detected ([Bradbury et al. 2005]; [Bourgis et al. 2008]; [Shi et al. 2008]; [Kovach et al. 2009]; [Sakthivel et al. 2009]).

Although aromatic varieties can be found in isozymic groups 1, 5 and 6, their frequency is much higher in group 5 ([Singh et al. 2000]). ([Glaszmann 1987, 1988]) reported that a high proportion of Myanmar varieties belonged to group 5 and that some of these were peculiar in terms of allelic combination, notably at isozyme locus *Amp3* on chromosome 6, as well as in terms of grain quality. Myanmar occupies a key geographic position at the interface between South and South-East Asia. Myanmar is geographically diverse with four recognizable regions (the Ayeyarwady delta, a coastal strip, the central dry plain and mountain ranges). Rice is an important crop for Myanmar, which has the highest per capita consumption of rice in the world: more than 210 kg per person per year. Rice cultivation covers eight million hectares (http://faostat.fao.org, 2009), mainly rainfed lowland rice (54% of the area), but also deepwater rice (24%) irrigated rice (18%), and upland rice (6%) (IRRI, 2002). Because of the interface position and ecological diversity of Myanmar, the genetic diversity of Myanmar rice varieties is expected to be high.

Studies on the diversity of sets of varieties from Myanmar have already been conducted using isozymes ([Khush et al. 2003]; [Tun 2006]) but seldom with molecular markers ([Yamanaka et al. 2011]) and never with a focus on aromatic accessions. Accessions from Myanmar were not widely represented in the worldwide genetic studies conducted so far ([Garris et al. 2005]; [Kovach et al. 2009]).

The objective of this study was to characterize the genetic diversity of a collection of Myanmar varieties, notably those belonging to group 5 and those known to be aromatic, and to relate it to the diversity observed in the *BADH2* gene.

## Methods

### Material

The 147 varieties from Myanmar we analyzed are listed in Table 1. A first subset of 96 accessions including 62 aromatic and 34 non-aromatic varieties originating from different geographical regions in Myanmar were provided by the Myanmar Seed Bank (“MSB” ID prefix). The isozyme group to which these accessions belonged was unknown. The second subset of 51 accessions including 3 aromatic, 45 non aromatic and 3 varieties with undetermined fragrance from Myanmar were provided by IRRI Genebank (“IRGC” ID prefix). According to the results of ([Khush et al. 2003]), all the members of the second subset belong to isozymic group 5. The aromatic/non aromatic quality of the varieties had already been determined by the Myanmar Seed Bank based on sensory tests. The sensory tests, based on leaf samples, were repeated for confirmation (Myo Myint, personal results) following the protocol of ([Sood and Siddiq 1978]).

**Table 1:** **List of the 147 Myanmar accessions assayed**

ID	Variety name	Region of origin	Ecosystem	EG	Structure	Aroma	Genotype	Genotype	Haplotype
					group		8 bp del.	3 bp ins.	
MSB_1158	BAHAN HMWE	Ayeyarwady Division	RL	u	1	Ar	P	A	H1
MSB_1774	BAY KYAR	Sagaing Division	RL	u	m	Ar	A	A	H12
MSB_3708	BAY KYAR PAW SAN	Yangon Division	RL	u	5B	Ar	A	P	H5
MSB_325	CHIN KAUK HNYIN	Sagaing Division	u	u	1	Ar	A	A	H7
MSB_1898	EMAHTA HMWE	Kachin State	RL	u	1	Ar	A	A	H7
MSB_2297	KAMAR KYI SAW	Mon State	RL	u	5B	Ar	A	P	H5
MSB_6071	KAUK HNYIN HMWE	Shan State	u	u	1	Ar	P	A	H1
MSB_6108	KAUK HNYIN NET	Shan State	u	u	5B	Ar	A	P	H5
MSB_6097	KAUK HNYIN PHYU	Shan State	u	u	5B	Ar	A	P	H5
MSB_371	KAUK HNYIN SAW	Kachin State	u	u	1	Ar	u	A	ns
IRGC_58069	KYET PAUNG	Semi-arid zone	u	5	5A	Ar	P	A	H1
MSB_235	KYET PAUNG SAN	Sagaing Division	RL	u	1	Ar	A	A	H7
MSB_1932	KYWET THWAR	Yangon Division	u	u	1	Ar	A	A	H10
MSB_7293	MAN AUNG KAUK YIN	Rakhine State	u	u	5B	Ar	A	A	H4
MSB_398	MEE DON YIN	Mandalay Division	u	u	5B	Ar	A	A	H3
MSB_829	MYA WAR NGA CHEIK	Sagaing Division	u	u	1	Ar	u	A	H6
MSB_2283	NAMATHALAY	Sagaing Division	u	u	5A	Ar	P	A	H1
MSB_1809	NAT PYI HMWE	Bago Division	u	u	5B	Ar	u	u	H4
MSB_1382	NGA BYA YIN KAUK HNYIN NET	Sagaing Division	u	u	5B	Ar	A	P	H5
MSB_1777	NGA CHEIK	Kachin State	u	u	5B	Ar	A	A	H4
MSB_5884	NGA CHEIK	Chin State	u	u	1	Ar	A	A	H13
MSB_6101	NGA CHEIK KAUK HNYIN	Shan State	u	u	1	Ar	A	A	ns
MSB_1790	NGA KYWE	Bago Division	RL	u	1	Ar	u	A	ns
MSB_56	NGA KYWE	Mon State	RL	u	5B	Ar	A	A	H2
MSB_1105	NGA KYWE (U TO)	Ayeyarwady Division	RL	u	5B	Ar	A	A	ns
MSB_1638	NGA KYWE NOTE	Bago Division	RL	u	5B	Ar	A	A	H4
MSB_348	NGA KYWE PHYU	Ayeyarwady Division	u	u	1	Ar	A	A	ns
MSB_352	NGA KYWE PHYU	Ayeyarwady Division	RL	u	1	Ar	A	A	H7
MSB_1114	NGA KYWE TAUNG PYAN	Ayeyarwady Division	RL	u	5B	Ar	A	A	ns
MSB_1799	NGA KYWE TAUNG PYAN	Yangon Division	RL	u	1	Ar	A	A	H2
MSB_1789	NGA KYWE YIN	Kayin State	RL	u	5B	Ar	A	P	H5
MSB_410	NGA KYWE YIN	Ayeyarwady Division	RL	u	5B	Ar	A	A	ns
MSB_1871	NGA PYA GYI	Mandalay Division	RL	u	5B	Ar	A	P	H5
IRGC_33552	PATHEIN NYUNT	Ayeyarwady delta zone	u	5	5B	Ar	A	P	H5
MSB_2579	PAW SAN BAY KYAR	Ayeyarwady Division	RL	u	5B	Ar	A	P	H5
MSB_2877	PAW SAN BAY KYAR	Ayeyarwady Division	RL	u	1	Ar	u	A	H7
MSB_2925	PAW SAN BAY KYAR	Ayeyarwady Division	RL	u	5B	Ar	u	A	ns
MSB_3163	PAW SAN BAY KYAR	u	RL	u	5B	Ar	u	P	ns
MSB_804	PAW SAN BAY KYAR	Ayeyarwady Division	RL	u	5B	Ar	u	P	H5
MSB_807	PAW SAN BAY KYAR	Ayeyarwady Division	RL	u	5B	Ar	A	A	H2
MSB_1207	PAW SAN HMWE	Ayeyarwady Division	u	u	5B	Ar	A	P	H5
MSB_1641	PAW SAN HMWE	Ayeyarwady Division	RL	u	1	Ar	A	A	ns
MSB_2082	PAW SAN HMWE	u	RL	u	1	Ar	u	A	ns
MSB_2502	PAW SAN HMWE	Ayeyarwady Division	RL	u	1	Ar	A	A	H6
MSB_2620	PAW SAN HMWE	Ayeyarwady Division	RL	u	5B	Ar	u	P	ns
MSB_5802	PAW SAN HMWE	u	RL	u	1	Ar	u	A	ns
MSB_6973	PAW SAN HMWE BAY KYAR	Rakhine State	RL	u	5B	Ar	u	P	H5
MSB_7005	PAW SAN HMWE KAUK YIN	Rakhine State	RL	u	5B	Ar	A	A	H2
MSB_2577	PAW SAN SHWE WAR	Ayeyarwady Division	u	u	5B	Ar	A	A	H2
MSB_2924	PAW SAN TAUNG PYAN HMWE	Yangon Division	u	u	5B	Ar	u	A	H2
MSB_1128	PAW SAN YIN	Ayeyarwady Division	RL	u	5B	Ar	A	P	H5
MSB_163	SABA NET	Mandalay Division	RL	u	1	Ar	A	A	ns
MSB_2261	SABA NET	Kayah State	RL	u	m	Ar	u	A	H6
MSB_1915	SABA NET KAUK YIN	Mandalay Division	I	u	5B	Ar	u	A	ns
MSB_682	SABANI HMWE	Tanitharyi Division	RL	u	1	Ar	P	A	H1
MSB_75	SHWE WAR KAUK HNYIN	Yangon Division	u	u	1	Ar	A	A	H2
MSB_1628	TAUNG PYAN HMWE	Kayin State	RL	u	1	Ar	A	A	H9
MSB_1621	TAUNG PYAN SA BA NET	Bago Division	RL	u	m	Ar	A	A	H9
MSB_6096	TAUNG YAR KAUK HNYIN	Shan State	u	u	1	Ar	A	A	H2
MSB_1786	THET NU SABA NET PYAN	Yangon Division	RL	u	5B	Ar	u	A	H4
MSB_1791	TYAUNG PYAN YIN	Ayeyarwady Division	RL	u	1	Ar	u	A	H1
MSB_1792	TYAUNG PYAN YIN	Bago Division	RL	u	5B	Ar	u	A	H4
IRGC_32293	YANGON SABA	Southern plain zone	u	5	5B	Ar	A	P	ns
MSB_788	YAR KAUK HNYIN	Shan State	RL	u	1	Ar	A	A	ns
MSB_2528	YWA LWE	Kachin State	RU	u	1	Ar	A	A	H7
MSB_311	AUNG LAN DAW	Bago Division	RL	u	1	Na	A	A	ns
IRGC_32959	BALUGUN KAUKKYI	Ayeyarwady delta zone	u	5	5B	Na	A	A	ns
MSB_537	BAW GYI	Ayeyarwady Division	RL	u	1	Na	A	A	ns
MSB_211	BU DO THE	Shan State	RU	u	1	Na	A	A	ns
MSB_769	BU RO	Kayin State	RL	u	1	Na	A	A	ns
MSB_2587	BU SABA	Tanitharyi Division	RL	u	1	Na	A	A	H14
IRGC_6804	D 44-1	Southern plain zone	u	5	5B	Na	A	A	H3
MSB_635	EPA TINE	Kayin State	RL	u	1	Na	A	A	ns
MSB_2512	KALA GYI NGA SEIN	Mon State	RL	u	1	Na	A	A	ns
MSB_498	KAUK KYI THEE DAT	Bago Division	RL	u	1	Na	A	A	ns
IRGC_33191	KAUKKYI MEEDON	Ayeyarwady delta zone	u	5	1	Na	A	A	ns
MSB_240	KHAO KHAN NEW	Shan State	RU	u	1	Na	A	A	ns
MSB_263	KHAO LONE KHONE	Shan State	RU	u	1	Na	A	A	ns
MSB_158	KHAO MON HAN YOUNG	Kayah State	RU	u	1	Na	A	A	ns
MSB_519	KHAO NWAN	Shan State	RU	u	1	Na	A	A	ns
MSB_218	KHAO SING SAUK	Shan State	RU	u	1	Na	A	A	ns
MSB_353	KYAR GALAY	Mon State	RL	u	1	Na	A	A	ns
MSB_728	KYAW KHAW	Kachin State	RU	u	1	Na	A	A	ns
MSB_230	KYEE MASHE	Mandalay Division	RL	u	1	Na	A	A	ns
IRGC_33280	KYEEARNI	Southern plain zone	u	5	5B	Na	A	A	ns
IRGC_33282	KYEEME	u	u	5	5B	Na	u	u	ns
IRGC_33283	KYEENI	Southern plain zone	u	5	5B	Na	A	A	ns
IRGC_33287	KYWEKYUT MEEDON	Ayeyarwady delta zone	u	5	m	Na	A	A	ns
IRGC_33301	LAWTHAWPHINME (E62-10)	Southern coastal zone	u	5	5B	Na	A	A	ns
IRGC_33304	LEIKKALAY MEEDON	Southern plain zone	u	5	5B	Na	A	A	H2
MSB_2560	LET TAW YWE BAW	Mon State	RL	u	1	Na	A	A	ns
MSB_577	LONE PHYU	Mon State	u	u	1	Na	A	A	ns
MSB_285	MA PO LAY	Ayeyarwady Division	RL	u	1	Na	A	A	ns
IRGC_33373	MAYZI	Western coastal zone	u	5	m	Na	A	A	ns
IRGC_33379	MEEDON YIN	Semi-arid zone	u	5	5B	Na	A	A	ns
IRGC_33380	MEEDON YOYO	Ayeyarwady delta zone	u	5	1	Na	A	A	ns
MSB_109	NAGA NEW	Rakhine State	RL	u	1	Na	A	A	ns
IRGC_58152	NGA KYWE GYI	Western coastal zone	u	5	m	Na	A	A	H6
MSB_404	NGA YUN WAR	Bago Division	RL	u	1	Na	A	A	ns
IRGC_33466	NGAKYEIN THEE SHAY	Western coastal zone	u	5	1	Na	A	A	ns
IRGC_33467	NGAKYWE	Ayeyarwady delta zone	u	5	m	Na	A	A	ns
IRGC_33468	NGAKYWE	Ayeyarwady delta zone	u	5	m	Na	A	A	ns
IRGC_33469	NGAKYWE	Ayeyarwady delta zone	u	5	5B	Na	A	A	ns
IRGC_11142	NGAKYWE TAUNG PYAN	Ayeyarwady delta zone	u	5	5B	Na	A	A	ns
IRGC_33478	NGA-KYWE YIN	Eastern plateau	u	5	5B	Na	A	A	H2
IRGC_58154	NGAYA PAUK	Western coastal zone	u	5	m	Na	A	A	ns
IRGC_33521	NGAYUN TAUNGPYAN	Southern plain zone	u	5	1	Na	A	A	ns
IRGC_58172	PATEEPU/OO PAUK	Southern plain zone	u	5	5A	Na	A	A	ns
IRGC_33551	PATHEIN NGAKYWE	Ayeyarwady delta zone	u	5	5B	Na	A	A	ns
IRGC_33590	POKEKYI	Southern plain zone	u	5	5B	Na	A	A	ns
IRGC_33596	PYAPON THEEDAT	Ayeyarwady delta zone	u	5	m	Na	A	A	ns
IRGC_33605	SABANET KAUKYIN	Semi-arid zone	u	5	5B	Na	A	A	ns
IRGC_58191	SAN KAR YAN THAE	Southern plain zone	u	5	6	Na	A	A	H2
MSB_234	SAN SHWE NI	Bago Division	RL	u	1	Na	A	A	ns
MSB_66	SHWE NI TWAT SUN	Yangon Division	RL	u	1	Na	A	A	ns
IRGC_33668	SHWEDINGA	Semi-arid zone	u	5	5B	Na	A	A	ns
IRGC_33704	SHWEWA	Ayeyarwady delta zone	u	5	5B	Na	A	A	ns
IRGC_33708	SHWEWA HNAN	Ayeyarwady delta zone	u	5	5B	Na	A	A	ns
IRGC_33709	SHWEWA KAMAKYI	Ayeyarwady delta zone	u	5	5B	Na	A	A	ns
IRGC_33706	SHWEWAGYI	Ayeyarwady delta zone	u	5	1	Na	A	A	ns
IRGC_33712	SHWEWAYIN	Southern plain zone	u	5	1	Na	A	A	ns
MSB_217	TADAUNG PO	Ayeyarwady Division	D	u	1	Na	A	A	ns
MSB_1403	TAR SAING	Kachin State	RU	u	1	Na	A	A	ns
IRGC_33745	TAUNGDI	Southern plain zone	u	5	5B	Na	A	A	ns
IRGC_33747	TAUNGGYI KAUKKYI	Eastern plateau zone	u	5	5B	Na	A	A	ns
IRGC_33752	TAUNGPYAN	Ayeyarwady delta zone	u	5	5B	Na	A	A	ns
IRGC_33755	TAUNGPYAN KAUKNGE	Ayeyarwady delta zone	u	5	5B	Na	A	A	ns
IRGC_33757	TAUNGPYANYIN	Southern plain zone	u	5	5B	Na	A	A	ns
IRGC_33749	TAUNGTI	Southern plain zone	u	5	5B	Na	A	A	ns
IRGC_33773	THATNU SABANET	Southern plain zone	u	5	5B	Na	A	A	ns
MSB_984	THEE DAT PYA PON	Ayeyarwady Division	RL	u	1	Na	A	A	ns
MSB_67	THEE HTAT NGA SEIN	Yangon Division	RL	u	1	Na	A	A	H6
MSB_647	THEE HTAT PIN KHINE	Sagaing Division	RL	u	5A	Na	A	A	H2
IRGC_33791	THIT PIN	Western coastal zone	u	5	m	Na	A	A	ns
MSB_65	THONE HNAN PWA	Yangon Division	RL	u	1	Na	A	A	ns
IRGC_33805	THONESATOE NGAKWE	Southern plain zone	u	5	5B	Na	A	A	ns
IRGC_33802	THONSATOE	Semi-arid zone	u	5	2	Na	A	A	ns
MSB_113	TIN TANE	Kachin State	RL	u	1	Na	A	A	ns
MSB_925	WA KHE MA HNAN KAR	Ayeyarwady Division	D	u	1	Na	A	A	ns
IRGC_33835	WUNKYAW	Southern plain zone	u	5	1	Na	A	A	ns
IRGC_58247	YAT SAUK SABA	Eastern plateau zone	u	5	m	Na	A	A	ns
MSB_91	YAY MA NAING MIN THAR GYI	Yangon Division	D	u	1	Na	A	A	H7
MSB_33	ZAWTIKA GYI	Yangon Division	RL	u	1	Na	A	A	ns
MSB_233	ZEIN YIN	Bago Division	RL	u	1	Na	A	A	ns
IRGC_33192	KAUKKYISAW	Southern coastal zone	u	5	5B	u	A	A	H6
IRGC_5946	NGAKYWE D 25-4	Southern plain zone	u	5	m	u	A	A	H6
IRGC_33888	YELAIK MEEDON	u	u	5	5B	u	A	A	H2

*RL* rainfed lowland, *RU* rainfed upland, *D* deepwater, *I* irrigated, *u* unknown, *EG* Enzymatic Group, *m* = admixed; *Ar* aromatic, *Na* non-aromatic;

Genotype: 8 bp del. = 8 bp deletion in exon 7 in comparison with Nipponbare sequence; 3 bp ins. = 3 bp insertion in exon 13 in comparison with Nipponbare sequence; *A* absent, *P* present, Haplotype: *ns* not sequenced.

We complemented this set with 80 varieties listed in Additional file 1: Table S1 extracted from a core collection representing the varietal group diversity of *Oryza sativa* for which the enzymatic group had already been determined ([Glaszmann et al. 1995]). This set, hereafter referred to as “reference set”, was composed of 20 accessions from group 1 (indica), 9 from group 2 (aus/boro), 31 from group 5 (sadri/basmati), and 20 from group 6 (japonica), so group 5 accessions were over-represented. The information on the aromatic/non-aromatic nature of these accessions came from [Kovach et al. (2009]) and, for those that were not tested by these authors, from breeders’ knowledge ( Additional file 1: Table S1). Those for which the information was not available were labeled “unknown” (u).

### Genotyping

DNA was extracted from one plant per accession using the MATAB method ([Risterucci et al. 2000]). A set of 19 simple sequence repeat (SSR) markers distributed on the 12 chromosomes was genotyped on the 147 Myanmar accessions and the 31 reference set accessions from group 5. The SSR markers are listed in Table 2. The other 49 reference set accessions had already been genotyped with the same markers (Courtois et al. unpublished results). The genotyping was performed according to the protocol of ([Roy et al. 1996]) implemented with the automated infrared fluorescence technology of LI-COR 3200 sequencers (Li-COR, Lincoln, Nebraska, USA) at CIRAD genotyping and robotics platform, Montpellier, France. Primer sequences were retrieved from the Gramene database (http://www.gramene.org). For a given SSR locus, the forward primer was designed with a 5'-end M13 tail (5'-CACGACGTTGTAAAACGAC-3'). The polymerase chain reaction (PCR) amplifications were performed in a Eppendorf Mastercycler (Eppendorf, Hamburg, Germany) on 25 ng of DNA in a final volume of 10 μl of buffer (10 mM Tris–HCl (pH 8), 100 mM KCl, and 0.5 mM MgCl2) containing 0.08 μM of the M13-tailed primer, 0.1 μM of the other primer, 200 μM of dNTP, 1 U of Taq DNA polymerase (Invitrogen, Carlsbad, California, USA), and 0.1 μM of M13 primer-fluorescent dye IR700 or IR800 (Eurofins-MWG, Ebersberg, Germany). The PCR programme included an initial denaturation cycle at 95°C for 4 min, 35 cycles at 94°C for 1 min, Tm for 1 min, and 72°C for 1 min, and a final elongation step at 72°C for 8 min. IR700 or IR800-labeled PCR products were diluted 7-fold and 5-fold respectively, subjected to electrophoresis in a 6.5% polyacrylamide gel and then sized by the infra red fluorescence scanning system of the sequencer. Allele calling was performed twice by two different persons based on five DNA pools of known allele size included in each gel and used as standards.

**Table 2:** **Characteristics of the 19 SSR loci including their repeat motif, the number of alleles per locus, and PIC value in the two rice collections (Myanmar and reference set)**

Marker	Chr.	SSR motif	Reference set accessions^1^		Myanmar collection^2^		Reference set group 5^3^		Myanmar group 5^4^	
			No of alleles	PIC	No of alleles	PIC	No of alleles	PIC	No of alleles	PIC
RM1	1	(GA)26	13	0.82	16	0.73	5	0.50	9	0.48
RM5	1	(GA)14	11	0.85	9	0.82	8	0.72	9	0.83
RM11	7	(GA)17	10	0.78	9	0.67	4	0.36	7	0.37
RM25	8	(GA)18	11	0.69	10	0.75	6	0.45	6	0.64
RM44	8	(GA)16	11	0.72	5	0.67	3	0.42	5	0.53
RM124	4	(TC)10	4	0.56	3	0.42	4	0.60	3	0.30
RM154	2	(GA)21	15	0.89	12	0.85	9	0.74	10	0.82
RM215	9	(CT)16	7	0.66	7	0.52	4	0.50	6	0.45
RM237	1	(CT)18	8	0.79	11	0.60	5	0.60	5	0.32
RM271	10	(GA)15	7	0.69	8	0.53	3	0.37	7	0.33
RM287	11	(GA)21	11	0.61	12	0.70	4	0.23	9	0.60
RM316	9	Complex	9	0.73	7	0.30	7	0.58	6	0.22
RM338	3	(CTT)6	3	0.40	2	0.24	2	0.17	2	0.36
RM431	1	(AG)16	10	0.75	9	0.67	5	0.47	7	0.62
RM447	8	(CTT)8	8	0.65	4	0.48	4	0.49	4	0.39
RM474	10	(AT)13	14	0.87	12	0.71	6	0.67	7	0.55
RM510	6	(GA)15	7	0.58	6	0.47	3	0.30	4	0.39
RM538	5	(GA)14	11	0.69	11	0.68	6	0.48	6	0.41
RM1227	12	(AG)15	8	0.77	6	0.72	7	0.74	6	0.67
Total			178		159		95		118	
Average			9.3	0.71	8.4	0.61	5.0	0.49	6.2	0.49

^1^ 80 accessions; ^2^ 147 accessions; ^3^ 31 accessions; ^4^ 51 accessions.

The 178 accessions were also genotyped with two other primer pairs, the first one to detect an 8 bp deletion and the second one to detect a 3 bp insertion in the coding sequence of the *BADH2* gene. The sequences of the two primer pairs are listed in Additional file 1: Table S2.

### Statistical analyses

The allele number, allele frequencies and polymorphism information content (PIC) of each marker were computed using PowerMarker version 3.25 ([Liu and Muse 2005]).

We used both a distance-based and a model-based approach to assess the genetic structure of the whole population (Myanmar accessions and the reference set). A dissimilarity matrix was computed using a shared allele index with DarWin software ([Perrier and Jacquemoud-Collet 2006]). An unweighted neighbor-joining (NJ) tree was built based on this dissimilarity matrix. The number of sub-populations, K, in the population was assessed in parallel using the software Structure v2.3 ([Pritchard et al. 2000]). The program was run with the following parameters: haploid data, possibility of admixture and correlated allelic frequencies. A run was composed of 100,000 burn-ins followed by 100,000 iterations. We ran 10 runs for each value of K, with K varying from 1 to 10. For each run, the percentages of admixture in the K sub-populations of all accessions were computed. An accession was discretely classified in a sub-population when the admixture percentage of the accession for the sub-population concerned was above 80%. Otherwise it was classified as admixed (m). To determine the most likely K value, we also used the criteria proposed by [Evanno et al. (2005]) related to the first and second order rates of change of the likelihood function with respect to K.

We used XLSTAT tree classification tools ([Breiman et al. 1984]) under Excel (http://www.xlstat.com/) to determine which markers or marker combinations best distinguished sub-populations.

The hierarchical distribution of the molecular variance within and between the sub-populations defined by Structure was assessed by analysis of molecular variance (AMOVA) with Arlequin ([Excoffier et al. 2006]). To evaluate the genetic differentiation between these populations, pair-wise F_ST_ ([Wright 1978]) were computed with the same software, using 1000 permutations to determine their significance.

### Sequencing

We sequenced the *BADH2* gene (gene Os08g32870 retrieved from Gramene; position in MSU v6 chromosome 8: 20,377,081 to 20,383,348 bp) in 91 accessions: 50 aromatic, 7 non-aromatic and 3 unknowns from the Myanmar collection, and 17 aromatic, 5 non-aromatic including Nipponbare, and 9 unknowns from the reference set (Table 1). The 12 primer pairs covering the whole *BADH2* gene listed in Additional file 1: Table S2 came from [Vanavichit et al. (2008]), [Kovach et al. (2009]) or Khin et al. (2012). Amplification products were purified and sequenced at GATC, Germany. The sequences, except that of Nipponbare, were deposited in the EMBL-Genbank database under the accession numbers [GenBank:JQ308346 to JQ308435].

Sequence quality control, alignment and nucleotide polymorphism detection were performed using Codon Code Aligner v1.6.3 (http://www.codoncode.com/index.htm) with minimum Phred scores set at 20. Polymorphism sites were numbered starting with position 1 for the A of the ATG of the first exon (position in MSU v6: 20,377,257) based on the annotation of [Chen et al. (2008]). Haplotypes were manually determined. Haplotype networks, representing unique alleles separated by mutational steps, were constructed with NETWORK v4.5 (http://www.fluxus-techno-logy.com) using the median-joining method ([Bandelt et al. 1999]), with equal weight for all sites and an eta-parameter of 0. Both single nucleotide polymorphism (SNP) and insertions/deletions (Indels) were used, with the Indels coded in biallelic form as SNPs.

## Results

### Genotyping

A total of 147 varieties from Myanmar and 80 varieties from the reference set were genotyped with 19 markers. The rate of missing data was 2.4%. The percentage of heterozygosity was 1.4%. The PIC of each marker in the different sets is listed in Table 2.

The number of alleles per marker and the PIC were generally slightly lower in the Myanmar collection than in the reference set. Focusing on group 5 accessions, differences between the two sets were also observed from marker to marker but the Myanmar collection was globally as diverse as the reference set.

We built an NJ tree and analyzed it using the accessions with known enzymatic grouping as references ( Additional file 1: Figure S1). A clear structure was revealed with one cluster of isozyme group 1 accessions, one cluster of isozyme group 2 accessions, one cluster of isozyme group 6 accessions, two clusters of isozyme group 5 accessions, and a few intermediates. The first cluster of isozyme group 5 accessions that we called 5A was mainly composed of varieties from South Asia (India, Nepal, Bhutan, and Pakistan), Iran and Madagascar, and one variety from Myanmar (Kyet Paung), and the second cluster that we called 5B was strictly composed of varieties from Myanmar. A few accessions among those said to belong to the Myanmar enzymatic group 5 did not cluster with the expected group: eight accessions in group 5 (Kaukkyi Meedon, Ngakyein Thee Shay, Ngakywe (IRGC 33468), Ngayun Taungpyan, Shwewagyi, Shewewayin, Wunkyaw, Meedon Yoyo) clustered with group 1 (as did two accessions in reference set group 5, T26 and Chote Dhan), two accessions clustered with group 2 (Thonsatoe, Yat Sauk Saba), one with group 6 (San Kar Yan Thae), and three additional accessions (Ngakywe D25-4, Nga Kywe Gyi, Ngakywe (IRGC 33467)) were intermediates between the groups. The Myanmar accessions for which the isozyme group was unknown, clustered massively with either group 1 (61 accessions) or group 5B (32 accessions); only two of them clustered with group 5A (Namathalay and Thee Htat Pin Khine), and one was located with the intermediates (Paw San Hwme (MSB 2923)).

**Figure 1:**
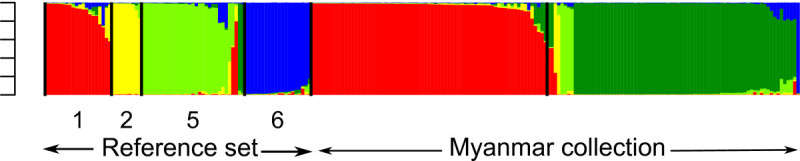
**Graph of estimated membership fraction for K = 5 (run with the highest likelihood).** The first part of the graph represents the reference set with a vertical black bar separating the enzymatic groups.The first part of the graph represents the reference set with a vertical bar separating the enzymatic groups. The second part of the graph includes the Myanmar accessions. The five different colors correspond to the five Structure groups. Enzymatic groups: 1 = indica, 2 = aus/boro, 5 = sadri/basmati; 6 = japonica ([Glaszmann, 1987]).

Based on Structure results and Evanno's criteria ( Additional file 1: Figure S2), the most likely number of sub-populations was five. We analyzed the results again based on the isozyme-based organization of the reference set. The five sub-populations detected corresponded to group 1, group 2, group 5A, group 5B and group 6 (Figure 1). The projection of Structure assignation for K = 5 on the NJ tree was in excellent agreement with the clusters identified, including for those genotypes from enzymatic group 5 which had appeared in an unexpected NJ tree cluster. Seventeen varieties (7.5%) were admixed.

Hierarchical analysis of molecular variance revealed a highly significant genetic differentiation among the five sub-populations with 45% of the variance due to differences among sub-populations and 55% due to difference within sub-populations. The pair-wise F_ST_ varied between 0.31 and 0.63, indicating high differentiation between sub-populations. Sub-population 5B showed a high and similar level of differentiation from group 1 (FST of 0.52), group 6 (0.50) and from the 5A sub-population (0.51) and an even higher level from group 2 (0.63). Sub-population 5A had moderate but similar levels of differentiation as group 1 (FST of 0.36) group 2 (0.34) and group 6 (0.31). These high F_ST_ were consistent with the strong structure revealed by the NJ tree.

The distinction between two sub-populations among accessions belonging to isozyme group 5 was unexpected. We used classification and regression tree methods ([Breiman et al. 1984]) to determine which markers best distinguished sub-population 5A from 5B. RM11, located on chromosome 7, enabled to *a posteriori* correctly predict 100% of the *a priori* grouping, with allele 140 present only in sub-population 5B and alleles 142, 144 and 148 present only in sub-population 5A. RM510 located on chromosome 6, had the same capacity with alleles 141 and 143, which were present only in sub-population 5B, and with alleles 131 and 139, which were present only in sub-population 5A. The next step was not as decisive, and three markers (RM237, RM 215 and RM338) located on chromosomes 1, 9 and 3 respectively were needed to predict 98% of the grouping.

We determined the aromatic/non-aromatic nature of most varieties from Myanmar (Table 1). A total of 65 of the 147 accessions (44%) were aromatic: 27 accessions among the 67 assigned to sub-population 1 (39%), two accessions among the 4 assigned to sub-population 5A (50%), 33 accessions among the 61 assigned to sub-population 5B (56%), and three admixed accessions (23%).

We genotyped two mutations (the 8 bp deletion in exon 7 and 3 bp insertion in exon 13) known to be associated with the presence of aroma. We tested the 147 Myanmar accessions and the 31 accessions from group 5 of the reference set (Tables 1 and Additional file 1: S1). As expected, all the accessions that had either the 8 bp deletion or the 3 bp insertion were aromatic. However, the reverse was not true. Thirty four aromatic varieties had neither of the mutations.

It should be noted that different accessions bearing the same name or root name, which generally indicates popular varieties, sometimes corresponded to distinct varietal types, differing in terms of aroma, functional mutation, or even assignment to a sub-population (e.g. Paw San Hmwe or Ngakywe)

### Sequencing

To explain the presence of accessions that were aromatic even though they did not have any of the known mutations, we sequenced the *BADH2* gene. Despite using high-fidelity long read DNA polymerase and specific PCR conditions, we were not able to consistently amplify the first two exons, probably because of the very high CG content of this zone. We amplified 4842 bp from intron 2 to 3' UTR, corresponding to 65% of the gene sequence and 78% of the coding sequence.

Among the 91 sequenced accessions listed in Tables 1 and Additional file 1: S1, we identified 63 polymorphisms corresponding to 54 SNPs, eight indels, and one SSR. Among the 63 polymorphisms, 26 were singletons almost exclusively found in two varieties: Kywet Thwar (9 specific polymorphisms), and Firooz (13 specific polymorphisms) with one in Thee Htat Pin Khine and two in Bu Saba. Nipponbare itself carried the minor allele at two SNPs, one of which was a singleton. Among the polymorphisms detected, very few were in the coding sequence: three SNPs adjacent to one 8 bp deletion in exon 7, one SNP in exon 9 (singleton in a non-aromatic accession), one SNP in exon 10 (singleton in an aromatic accession), and one 3 bp insertion in exon 13. One SNP, one 4 bp insertion (singleton in a non-aromatic accession) and one 43 bp deletion were located in exon 15 in the 3’UTR region based on the MSU annotation, but, based on the annotation by ( [Chen et al. 2008]) were located after the end of the gene.

**Figure 2:**
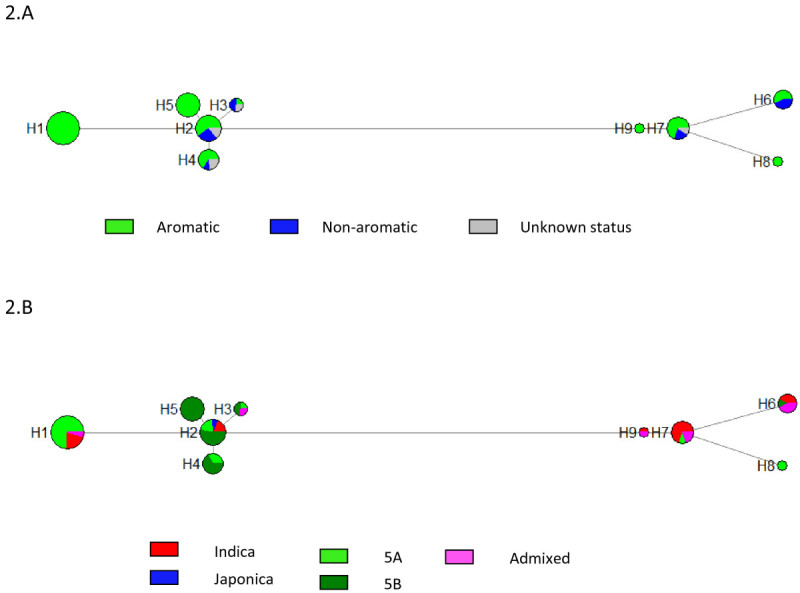
**Haplotype network for the gene** ***BADH2*** **Each node represents a haplotype, its size being proportional to its frequency.** **2A**. Distribution according to the aromatic nature of the accessions Green: aromatic; blue: non-aromatic; gray: unknown **2B**. Distribution according to Structure groups Red: 1 (indica); blue: 6 (japonica); light green = 5A; dark green = 5B; violet: admixed.

After eliminating the singletons and the SSR, we obtained 36 polymorphisms representing 15 haplotypes (Table 3). Six haplotypes were variety specific. Again, two corresponded to Kywet Thwar (H10) and Firooz (H11). The other four corresponded to Bay Kyar (H12), which appeared to have recombined at the end of the sequence, Nga Cheik (H13), Bu Saba (H14) and Nipponbare (H15). The remaining nine haplotypes were used to draw the haplotype network (Figure 2A and B). Two groups of haplotypes were detected (H1 to H5 and H6 to H9). In the first group, H1 included only aromatic accessions that carried the well known 3 SNPS −8 bp deletion polymorphism. H5 was also strictly composed of aromatic varieties that carried the 3 bp insertion which appeared to be specific to Myanmar accessions. The other haplotypes in the first group, which did not carry mutations in the coding sequence, were composed of a mixture of aromatic and non-aromatic accessions. In the second group, mutations in the coding sequence were found only at the very end of the last exon and did not correlate with the presence of fragrance (composite haplotypes). Few haplotypes were specific to a structure group; H8 was specific to 5A accessions (but included only two accessions), and H5 was strictly specific to 5B accessions.

**Table 3:** **Position of the 36 polymorphisms detected in the 91 accessions sequenced, after eliminating 26 singletons and one SSR, and structure of the 15 haplotypes observed**

Pos.	Zone	P	Nature	Freq.	Cons	H1	H2	H3	H4	H5	H6	H7	H8	H9	H10	H11	H12	H13	H14	H15
695	I2	snp	nc	2.2	C	C	C	C	C	C	C	C	C	C	C	C	G	G	C	C
825	I2	snp	nc	7.7	C	C	C	C	C	C	T	C	C	C	C	C	C	C	C	C
829	I2	snp	nc	3.3	T	T	T	T	T	T	T	T	T	T	T	T	C	C	C	T
839	I2	indel	nc	29.7	0	0	0	0	0	0	2	2	2	2	2	2	2	2	2	2
876	I2	snp	nc	2.2	A	A	A	A	A	A	A	A	G	A	A	A	A	A	A	A
915	I2	snp	nc	25.3	C	C	C	C	C	C	T	T	T	T	T	T	C	C	C	C
918	I2	snp	nc	2.2	T	T	T	T	T	T	T	T	C	T	T	T	T	T	T	T
1082	I2	snp	nc	21.9	T	T	T	T	T	T	C	C	C	T	T	T	T	T	T	T
1112	I2	snp	nc	25.3	A	A	A	A	A	A	T	T	T	T	T	T	A	A	A	A
1113	I2	indel	nc	25.3	3	3	3	3	3	3	0	0	0	0	0	0	3	3	3	3
1316	I2	snp	nc	7.7	G	G	G	G	G	G	A	G	G	G	G	G	G	G	G	G
1340	I2	snp	nc	23.1	C	C	C	C	C	C	G	G	G	G	C	C	C	C	C	C
1819	I4	snp	nc	25.3	T	T	T	T	T	T	A	A	A	A	A	A	T	T	T	T
2142	I4	snp	nc	26.4	T	T	T	T	T	T	A	A	A	A	A	A	T	T	T	A
2144	I4	snp	nc	8.8	T	T	T	T	T	T	A	T	T	T	T	A	T	T	T	T
2205	I4	snp	nc	26.4	A	A	A	A	A	A	G	G	G	G	G	G	G	A	A	A
2219	I4	snp	nc	25.3	G	G	G	G	G	G	A	A	A	A	A	A	G	G	G	G
2889	E7	snp	c	25.3	A	T	A	A	A	A	A	A	A	A	A	A	A	A	A	A
2891	E7	snp	c	25.3	A	T	A	A	A	A	A	A	A	A	A	A	A	A	A	A
2893	E7	snp	c	24.2	A	G	A	A	A	A	A	A	A	A	A	A	A	A	A	A
2894	E7	indel	c	25.3	8	0	8	8	8	8	8	8	8	8	8	8	8	8	8	8
3003	I7	indel	nc	7.7	T	T	T	T	T	T	0	T	T	T	T	T	T	T	T	T
3031	I7	snp	nc	3.3	G	G	G	G	G	G	G	G	G	G	G	G	T	T	T	G
3219	I8	snp	nc	12.1	C	C	C	C	T	C	C	C	C	C	C	C	T	T	C	C
3220	I8	snp	nc	25.3	G	G	G	G	G	G	A	A	A	A	A	A	G	G	G	G
3372	I8	snp	nc	25.3	C	C	C	C	C	C	G	G	G	G	G	G	C	C	C	C
3467	I8	snp	nc	25.3	T	T	T	T	T	T	C	C	C	C	C	C	T	T	T	T
3988	I9	snp	nc	2.2	T	T	T	T	T	T	T	T	T	T	G	G	T	T	T	T
4423	I10	snp	nc	2.2	C	C	C	C	C	C	C	C	C	C	G	G	C	C	C	C
4452	I10	snp	nc	2.2	A	A	A	A	A	A	A	A	A	A	A	A	G	G	A	A
5233	E13	indel	c	14.3	0	0	0	0	0	3	0	0	0	0	0	0	0	0	0	0
5491	I13	snp	nc	2.2	A	A	A	A	A	A	A	A	T	A	A	A	A	A	A	A
5531	I13	snp	nc	29.7	G	G	G	G	G	G	A	A	A	A	A	A	A	A	A	A
5688	I14	snp	nc	4.4	A	A	A	G	A	A	A	A	A	A	A	A	A	A	A	A
6049	3'UTR	snp	nc	3.3	T	T	T	T	T	T	T	T	T	T	A	T	T	A	A	T
6086	3'UTR	indel	nc	24.2	43	43	43	43	43	43	0	0	0	0	43	43	0	43	43	43
Aroma						Ar	M	M	M	Ar	M	M	Ar	Ar	Ar	Na	Ar	Ar	Na	Na
No acc.						23	15	4	9	13	7	10	2	2	1	1	1	1	1	1
Myanmar						6	13	2	6	13	7	7	0	2	1	0	1	1	1	0

Pos. = position of the polymorphism (position 1 for the A of the ATG of the first exon corresponding to position 20,377,257 in MSU v6). Indels of more than one bp: site 839: 2 = TT; site 1113: 3 = TTA; site 2894: 8 = ATTATGGC; site 5233: 3 = TTA; site 6086: 43 = GTTCTCTCCGTATCGGCTTGTGGTGTTTCAACCTTAAGACC.

Zone: I = intron; E = exon; Nature: nc = non coding; c = coding; Ar = aromatic; Na = non-aromatic; M = some accessions aromatic and other accessions non aromatic.

Cons = consensus sequence.

No acc. = number of accessions carrying the haplotype among the 91 sequenced; Myanmar = number of accessions from Myanmar carrying the haplotype among the 60 sequenced.

Correspondence with the haplotypes from ([Kovach et al. 2009]): H2 = wild type; H1 = *badh2.1*; H5 = *badh2.8*; H15 = Nipponbare haplotype.

A total of 24 accessions from Myanmar were aromatic but did not carry any mutations in the coding sequence of the segments sequenced in this study. They belonged to different haplotypes and different varietal groups (12 from group 1, 9 from group 5B, and 3 admixed). In addition, the three accessions from Madagascar belonging to group 5A (Kiriminy 1133, Kiriminy de 4 mois, and Kiriminy type Bengaly) did not carry any mutation.

## Discussion

We analyzed the genetic diversity of 147 accessions from Myanmar at neutral markers as well as the *BADH2* gene*,* which is responsible for aroma.

Based on isozymes, ([Khush et al. 2003]) classified the 1354 accessions from Myanmar stored in the IRRI genebank and found 1115 (82.4%) belonging to group 1 (indica), 17 (1.3%) to group 2, 52 (3.8%) to group 5, 30 (2.2%) to group 6 (japonica) and 140 (10.3%) to group 0 (intermediates). In comparison with this representation in the reference world collection, our set clearly over-represented group 5 accessions and under-represented japonica and intermediates, in accordance with our focus on aromatic rices.

Our analyses led to unexpected and original results, both in terms of varietal groups and distribution of aromatic varieties.

The results of Structure and of the distance-based NJ tree clearly separated group 5 accessions into two sub-groups. These two sub-groups carry diagnostic alleles on at least two SSR loci located on different chromosomes, eliminating the possibility that this could result from miscoding. The accessions in group 5A originate from a range of countries along the Himalayan foothills, while the accessions in group 5B appear to be limited to Myanmar. The fact that this group had not been identified previously is probably due to the small number of Myanmar accessions in earlier studies. This result shows the interest of broadening the samples analyzed with molecular markers to obtain a finer view of the genetic structure of *O. sativa*. The broadest systematic study to date remains the initial isozyme survey reported in 1987–1988. While proposing group 5 as a new group comprising varieties previously considered to be Indicas, the author of that study commented on the loose coherence of this group with a geographic cline placing Myanmar varieties at the eastern edge, with a near-specific allele at locus *Amp3*; yet this was the only locus with this pattern, providing insufficient grounds for distinguishing sub-groups on the basis of multi-locus linkage disequilibrium. The SSR survey of 3000 accessions in the Generation Challenge Programme and the sequencing of 10000 accessions by Beijing Genomics Institute and IRRI will undoubtedly fill this gap (McNally, IRRI, personal communication).

The position of *Amp3* has not yet been clearly defined on the rice genome; based on recombination mapping, it should be located between 5.5 and 7.5 Mb on chromosome 6, but a clear association with an annotated gene in the region remains to be established. RM510, whose polymorphism clearly separates 5A from 5B, is located on chromosome 6, in position 2.8 Mb, i.e. approximately 3 to 5 Mb from *Amp3*; linkage disequilibrium between two loci (*Amp3* and *Est2*) on chromosome 6 was highlighted long ago, as was its relation to varietal classification ([Glaszmann 1988]).

Among the Myanmar accessions, a very high proportion (44%) is aromatic. This high proportion was expected because our sampling focused on aromatic accessions. What is more surprising is the very high proportion of aromatic accession in group 1. The proportion (39%) is almost as high as in groups 5A and 5B (50 and 56%). Low yield is the main concern in aromatic varieties belonging to group 5 ( [Singh et al. 2000]), while rice top yielders in tropical irrigated conditions are generally indica varieties. The development of new aromatic varieties with an indica background is difficult using varieties from group 5 as donors because of inter-group compatibility problems. The identification of many new aromatic varieties belonging to group 1 may considerably broaden the range of varieties that breeders can use.

We sequenced part of the *BADH2* gene in most of the aromatic accessions of our sample. The diversity of the gene has already been studied in depth by ([Kovach et al. 2009]), who identified 10 aroma-associated alleles. Among the polymorphisms affecting the coding sequence in our sample, we found two mutations, the 3 SNPs/8 bp deletion (*badh2.1*) and 3 bp insertion (*badh2.8*) already identified by these authors. But the 3 bp insertion was present at a much higher frequency in our sample. It was recently demonstrated that this insertion did not modify the gene expression but the addition of a tyrosine to the peptide interfered with cofactor NAD + binding, lowered enzyme activity and led to accumulation of acetyl-pyrroline, although at a lower level than in accessions carrying the 8 bp deletion ( [Vanavichit and Yoshihashi 2010], [Wongpanya et al. 2011], [Myint et al. 2012]). We found one additional deletion of 43 bp at the very end of the gene in 22 accessions that was specific to Myanmar accessions. It is unclear whether this mutation is in the 3’UTR or outside the gene since the two annotations we used differed on exactly where exon 15 ends. However, the mutation does not control the production of aroma since the group includes both aromatic and non-aromatic accessions.

The 3 bp insertion is typical of Myanmar aromatic accessions belonging to group 5B. It is present in the most popular and widely grown aromatic varieties such as Paw San Hmwe and its declinations (Paw San Yin, Paw San Bay Kyar). The same situation was encountered among Chinese accessions with a specific allele that has not yet been found elsewhere ([Shi et al. 2008]). This suggests that the trait was selected on several occasions. While the 8 bp deletion was quite successful, since it is encountered in a large diversity of countries from Madagascar to China and in various genetic backgrounds (isozyme group 5A mostly but also groups 1 and 6), the 3 bp insertion appears to have remained more localized, i.e. limited to Myanmar and group 5B. This may be due to limited past varietal exchanges between Myanmar and neighboring countries but this hypothesis is unlikely since the 8 bp deletion was found in some of the indica aromatic accessions from Myanmar. It may result from the presence of this allele in less interesting backgrounds than those of the successful 8 bp deletion, or from a lower expression of the aroma due to the different nature of the mutations (amino acid addition in the case of the 3 bp insertion allele versus stop codon in the case of the 8 bp deletion allele).

Several aromatic accessions did not carry any mutation in the coding segments we sequenced. The reality of their aromatic nature can thus be questioned. Simple and fast methods such as the sniffing method or the one proposed by ([Sood and Siddiq 1978]) are commonly used and have been shown to be in very good agreement with gas chromatography ([Lorieux et al. 1996]) but false positives and false negatives (because of the transient nature of aroma expression) are known to occur. In addition, the link between aroma and acetyl-pyrroline is not always direct. Some varieties can be aromatic despite a low level of acetyl-pyrroline, probably because of higher levels of other compounds ([Sakthivel et al. 2009]). The Kiriminy varieties from Madagascar (group 5A) that do not carry the 8 bp deletion mutation are nevertheless said to be aromatic (N. Ahmadi, Cirad, personal communication) but whether the aroma comes from acetyl-pyrroline or from another compound has not yet been determined.

Assuming that the great majority of those accessions are indeed aromatic, the mutations are located either in the unsequenced coding part or in the promoter. However, only two rare mutations (singletons), both specific to tropical japonicas, were found by ([Kovach et al. 2009]) in the first and second exons that we did not sequence. ([Shi et al. 2008]) found a 7 bp deletion in exon 2 but the deletion appeared to be specific to Chinese accessions. The possibility that the functional mutation in those aromatic accessions differs from those already described cannot be ignored. A few accessions that did not have functional *BADH2* mutation that could explain their aromatic nature were also detected in previous studies ([Kovach et al. 2009]; [Sakthivel et al. 2009]). ([Fitzgerald et al. 2008]) postulated that production of acetyl pyroline could be driven by alleles at two different genes and not only by different alleles of *BADH2*. Rice has indeed a second *BADH1* gene located on chromosome 4 that codes for the *BADH* enzyme and acts in a similar way to *BADH2* but is regulated differently. A minor QTL for aroma l was detected by Lorieux et al. (2000) on the same region of chromosome 4. ([Singh et al. 2010]) found a haplotype of *BADH1* associated with aroma. However, they also showed that the mutation in *BADH2* (whose expression is constitutive) was a primary requirement and that *BADH1* mostly appeared to modify aroma intensity. But the acetyl-pyrroline biosynthesis pathway is complex and mutations in other genes than *BADH* paralogues may be responsible for the aroma, as suggested by ([Sakthivel et al. 2009]). The development of mapping populations involving accessions that do not carry known mutations in *BADH2* as a parent could help solve this issue.

## Conclusion

With one new group and the new alleles found in Myanmar, our study illustrates that the Himalayan foothills contain series of non-Indica and non-Japonica varietal types that bear novel variations for useful traits. These new alleles can be of interest for both local and more general plant breeding programs.

## Electronic supplementary material

Additional file 1 : **Table S1.** List of the 80 varieties in the reference set assayed. Table S2: List of the primers used to amplify segments of the *BADH2* gene. Figure S1: NJ tree representing the relative position of Myanmar accessions and the reference set. Figure S2: Evolution of the criteria that enabled detection of the likely number of subpopulations (K) in the 227 accessions using 19 SSR markers for K values varying from 1 to 10. (PDF 189 KB)

Below are the links to the authors’ original submitted files for images.

Authors’ original file for figure 1

Authors’ original file for figure 2

## References

[CR1] Bandelt HJ, Forster P, Rohl A (1999). Median-joining networks for inferring intraspecic phylogenies. Mol Biol Evol.

[CR2] Bourgis F, Guyot R, Gherbi H, Tailliez AI, Salse J, Lorieux M, Delseny GA (2008). Characterization of the major fragrance gene from an aromatic japonica rice and analysis of its diversity in Asian cultivated rice. Theor Appl Genet.

[CR3] Bradbury LMT, Fitzgerald TL, Henry RJ, Jin Q, Waters DLE (2005). The gene for fragrance in rice. Plant Biotechnol J.

[CR4] Bradbury LMT, Gillies SA, Brushett DJ, Waters DLE, Henry RJ (2008). Inactivation of an aminoaldehyde dehydrogenase is responsible for fragrance in rice. Plant Mol Biol.

[CR5] Breiman L, Friedman JH, Olshen R, Stone CJ (1984). Classification and regression trees.

[CR6] Buttery RG, Ling LC, Juliano BO (1982). 2-Acetyl-1-pyrroline: an important aroma component of cooked rice. Chem Ind (London).

[CR7] Chen S, Yang Y, Shi W, Ji Q, He F, Zhang Z, Cheng Z, Liu X, Xu M (2008). Badh2, encoding betaine aldehyde dehydrogenase, inhibits the biosynthesis of 2-acetyl-1-pyrroline, a major component in rice fragrance. Plant Cell.

[CR8] Evanno G, Regnaut S, Goudet J (2005). Detecting the number of clusters of individuals using the software STRUCTURE: a simulation study. Mol Ecol.

[CR9] Excoffier L, Laval G, Schneider S (2006). Arlequin v3.1. An integrated software for population genetic data analysis.

[CR10] Fitzgerald MA, Sackville Hamilton NR, Calingacion MN, Verhoeven HA, Butardo VM (2008). Is there a second fragrance gene in rice?. Plant Biotechnol J.

[CR11] Garris AJ, Tai TH, Coburn J, Kresovich S, McCouch S (2005). Genetic structure and diversity of O. sativa. Genetics.

[CR12] Gay F, Maraval I, Roques S, Gunata Z, Boulanger R, Audebert A, Mestres C (2010). Effect of salinity on yield and 2-acetyl-1-pyrroline content in the grains of three fragrant rice cultivars in Camargue. Field Crop Res.

[CR13] Glaszmann J-C (1987). Isozymes and classification of Asian rice varieties. Theor Appl Genet.

[CR14] Glaszmann J-C (1988). Geographic pattern of variation among Asian native rice cultivars based on fifteen isozyme loci. Genome.

[CR15] Glaszmann J-C, Arraudeau M (1986). Rice plant type variation: Japonica-Javanica relationships. Rice Genet Newsl.

[CR16] Glaszmann J-C, Mew T, Hibino H, Kim CK, Mew TI, Vera Cruz CH, Notteghem J-L, Bonman JM (1995). Molecular variation as a diverse source of disease resistance in cultivated rice. Rice Genetics III.

[CR17] Juliano BO (2007). Rice chemistry and quality.

[CR18] Kato S, Kosaha H, Hara S (1928). On the affinity of rice varieties as shown by the fertility of rice plants. Bull Sci Central Agric Kyushu Imperial Univ.

[CR19] Khush GS, Brar D, Virk PS, Tang SX, Malik SS, Busto GA, Lee YT, McNally R, Trinh LN, Jiang Y, Shat MAM (2003). Classifying rice germplasm by isozyme polymorphism and origin of cultivated rice. IRRI Discussion Paper 46.

[CR20] Kovach MJ, Calingacion MN, Fitzgerald MA, McCouch SR (2009). The origin and evolution of fragrance in rice. PNAS.

[CR21] Liu K, Muse SV (2005). PowerMarker: Integrated analysis environment for genetic marker data. Bioinformatics.

[CR22] Lorieux M, Petrov M, Huang N, Guiderdoni E, Ghesquière A (1996). Aroma in rice: genetic analysis of quantitative trait. Theor Appl Genet.

[CR23] Matsuo T (1952). Genecological studies on cultivated rice. Bull Natl Inst Agric Sci Jpn.

[CR24] Myint KM, Arikit S, Wanchana S, Yoshihashi T, Choowongkomon K, Vanavichit A (2012). A PCR-based marker for a locus conferring the aroma in Myanmar rice (Oryza sativa L.). Theor Appl Genet.

[CR25] Niu X, Tang W, Huang W, Ren G, Wang Q, Luo D, Xiao Y, Yang S, Wang F, Lu BR, Gao F, Lu T, Liu Y (2008). RNAi-directed down-regulation of OsBADH2 results in aroma (2-acetyl-1-pyrroline) production in rice (Oryza sativa L.). BMC Plant Biol.

[CR26] Oka HI (1958). Varietal variation and classification of cultivated rice. Ind J Genet Plant Breed.

[CR27] Perrier X, Jacquemoud-Collet JP (2006). DARwin software.

[CR28] Petrov M, Danzart M, Giampaoli P, Fayre J, Richard H (1996). Rice aroma analysis. Discrimination between a scented and non-scented rice. Sci Aliment.

[CR29] Pritchard JK, Stephens M, Donnelly P (2000). Inference of population structure using multilocus genotype data. Genetics.

[CR30] Risterucci AM, Grivet L, N'Goran JAK, Pieretti I, Flament MH, Lanaud C (2000). A high density linkage map of Theobroma cacao L. Theor Appl Genet.

[CR31] Roy R, Steffens DL, Gratside B, Jang GY, Brumbaugh JA (1996). Producing STR locus patterns from bloodstains and other forensic samples using an infrared fluorescent automated DNA sequencer. J Forensic Sci.

[CR32] Sakthivel K, Sundaram RM, Rani NS, Balachandran SM, Neereja CN (2009). Genetic and molecular basis of fragrance in rice. Biotechnol Adv.

[CR33] Shi W, Yang Y, Chen S, Xu M (2008). Discovery of a new fragrance allele and the development of functional markers for the breeding of fragrant rice varieties. Mol Breed.

[CR34] Singh RK, Singh US, Khush GS (2000). Aromatic rices.

[CR35] Singh A, Singh PK, Singh R, Pandit A, Mahato AK, Gupta DK, Tyagi K, Singh AK, Singh NK, Sharma TR (2010). SNP haplotypes of the BADH1 gene and their association with aroma in rice (O. sativa L.). Mol Breed.

[CR36] Sood BG, Siddiq EA (1978). A rapid technique for scent determination in rice. Indian J Genet Plant Breed.

[CR37] Tun YT (2006). Characterization of Myanmar rice landraces based on analyses of genetic diversity of various traits. PhD thesis.

[CR38] Unnevher LJ, Duff B, Juliano BO (1992). Consumer demand for rice grain quality.

[CR39] Vanavichit A, Yoshihashi T (2010). Molecular aspects of fragrance and aroma in rice. Adv Bot Res.

[CR40] Vanavichit A, Tragoonrung S, Toojinda T, Wanchana S, Kamolsukyunyong W (2008). Transgenic rice plants with reduced expression of Os2AP and elevated levels of 2-acetyl-1-pyrroline. US patent.

[CR41] Wongpanya R, Boonyalai N, Thammachuchourat N, Horata N, Arikit S, Myint KM, Vanavichit A, Choowongkomon K (2011). Biochemical and enzymatic study of rice BADH wild-type and mutants: an insight into fragrance in rice. Protein J.

[CR42] Wright S (1978). Evolution and the genetics of populations. Variability within and among natural population.

[CR43] Yamanaka S, Jatoi SA, Yi SS, Kothari SL, Htut T, Watanabe KN (2011). Genetic diversity of Myanmar rice and their implementation on management methods. Afr J Biotechnol.

