# Supplementary material for: Specific patterns of genetic diversity among aromatic rice varieties in Myanmar
Source: Rice (N Y). 2012 Aug 1;5:20. doi: 10.1186/1939-8433-5-20 (PMC5520840; doi:10.1186/1939-8433-5-20)
Supplement: Supplementary file 1 — Additional file 1 : Table S1. List of the 80 varieties in the reference set assayed. Table S2: List of the primers used to amplify segments of the BADH2 gene. Figure S1: NJ tree representing the relative position of Myanmar accessions and the reference set. Figure S2: Evolution of the criteria that enabled detection of the likely number of subpopulations (K) in the 227 accessions using 19 SSR markers for K values varying from 1 to 10. (PDF 189 KB) [file 12284_2011_16_MOESM1_ESM.pdf]

Supplementary Table 1: List of the 80 varieties in the reference set assayed

ID	Variety name	Country	Isozyme group	SSR genotyping	Structure group	Aroma	Genotype 8 bp del.	Genotype 3 bp ins.	Haplotype
IRGC_32380	ABRI	Bhutan	5	YES	5A	u	A	A	H2
IRGC_12386	ARC 10177	India	2	NO	2	Na	u	u	ns
IRGC_12485	ARC 10497	India	5	YES	5A	u	A	A	H4
IRGC_42469	ARC 13829	India	5	YES	5A	u	A	A	H3
IRGC_12331	ARC 7229	India	5	NO	m	Na	u	u	H3
IRGC_6267	ASD 1	India	1	NO	1	Na	u	u	ns
IRGC_7017	AUS 143	India	2	YES	2	Na	u	u	ns
IRGC_328	AZUCENA	Philippines	6	NO	6	Ar	P	A	ns
IRGC_27509	BARAN BORO	Bangladesh	2	NO	2	Na	u	u	ns
IRGC_27798	BASMATI 1	Pakistan	5	YES	5A	Ar	P	A	H1
IRGC_53637	BASMATI 217	India	5	YES	5A	Ar	P	A	H1
IRGC_6426	BASMATI 370	Pakistan	5	NO	5A	Ar	P	A	H1
IRGC_58881	BASMATI LAMO	Nepal	5	YES	5A	Ar	P	A	H1
IRGC_40275	BLACK GORA	India	2	NO	2	Na	u	u	ns
IRGC_50448	CANELA DE FERRO	Brazil	6	NO	6	Na	u	u	ns
IRGC_27869	CHAHORA 144	Pakistan	5	YES	5A	Ar	P	A	H1
IRGC_58930	CHHOTE DHAN	Nepal	5	YES	m	Na	A	A	H7
IRGC_17052	CHUAN 4	Taiwan	6	NO	6	Na	u	u	ns
IRGC_43372	CICIH BETON	Indonesia	6	NO	6	Na	u	u	ns
IRGC_10658	CUBA 65	Cuba	6	NO	6	Na	u	u	ns
IRGC_5857	DA 13	Bangladesh	5	YES	5A	Ar	P	A	H1
IRGC_12880	DOM SOFID	Iran	5	YES	5A	Ar	P	A	H1
IRGC_12881	DOM ZARD	Iran	5	YES	5A	Ar	P	A	H1
IRGC_32292	DOMSIAH	Iran	5	YES	5A	Ar	P	A	H1
IRGC_26011	DOURADO PRECOCE	Brazil	6	NO	6	Na	u	u	ns
IRGC_10984	FANDRAPOTSY 104	Madagascar	1	NO	m	Na	u	u	ns
IRGC_39261	FIROOZ	Iran	5	YES	5A	Na	A	A	H2
IRGC_32303	GHARIB	Iran	5	YES	m	Ar	P	A	H1
IRGC_43397	GOTAK GATIK	Indonesia	6	NO	6	Na	u	u	ns
IRGC_33085	GWA NGASEIN	Myanmar	1	NO	1	Na	u	u	ns
IRGC_4122	IGUAPE CATETO	Brazil	6	NO	6	Na	u	u	ns

ID	Variety name	Country	Isozyme group	SSR genotyping	Structure group	Aroma	Genotype 8 bp del.	Genotype 3 bp ins.	Haplotype
IRGC_33130	INDANE	Myanmar	6	NO	6	Na	u	u	ns
CIRAD_8351	IR 64	Philippines	1	NO	1	Na	A	A	ns
IRGC_10320	IR 8	Philippines	1	NO	1	Na	u	u	ns
IRGC_28508	IRAT 13	Côte d'Ivoire	6	NO	6	Na	u	u	ns
IRGC_27516	JAGLI BORO	Bangladesh	2	NO	2	Na	u	u	ns
IRGC_9091	JC 1	India	5	YES	5A	Ar	P	A	H1
IRGC_9060	JC 101	India	5	YES	5A	u	P	A	H1
IRGC_9070	JC 149	India	5	YES	5A	u	P	A	H1
IRGC_27967	JHONA 26	Pakistan	2	NO	2	Na	u	u	ns
IRGC_6618	KARKATI 87	Bangladesh	1	NO	1	Na	u	u	ns
IRGC_117617	KASALATH	India	2	NO	2	Na	u	u	ns
IRGC_33188	KAUKKYI ANI	Myanmar	5	NO	5B	u	u	u	ns
IRGC_27748	KHAO DAWK MALI 105	Thailand	1	NO	1	Ar	P	A	H1
IRGC_23423	KHAO KAP XANG	Laos	6	NO	6	Na	u	u	ns
IRGC_23364	KINANDANG PATONG	Philippines	6	NO	6	Na	u	u	ns
CIRAD_5109	KIRIMINY 1133	Madagascar	5	YES	5A	Ar	A	A	H8
CIRAD_5110	KIRIMINY DE 4 MOIS	Madagascar	5	YES	5A	Ar	A	A	H8
CIRAD_5112	KIRIMINY TYPE BENGALY	Madagascar	5	YES	5A	Ar	A	A	H7
IRGC_11010	MAINTIMOLOTSY 1226	Madagascar	6	NO	6	Na	u	u	ns
IRGC_6087	MAKALIOKA 34	Madagascar	1	NO	1	Na	u	u	ns
IRGC_8182	MALAGKIT PIRURUTONG	Philippines	6	NO	6	Na	u	u	ns
IRGC_23973	MANA MURI	Nepal	5	YES	5A	u	A	A	H4
IRGC_12883	MEHR	Iran	5	YES	5A	u	P	A	H1
IRGC_12048	MOROBEREKAN	Guinea-Conakry	6	NO	6	Na	u	u	ns
IRGC_6298	N 12	India	5	YES	5A	Ar	P	A	H1
IRGC_6264	N 22	India	2	NO	2	Na	u	u	ns
IRGC_11462	NAM SA GUI 19	Thailand	1	NO	1	Na	u	u	ns
IRGC_191	NHTA 10	India	6	NO	6	Na	u	u	ns
IRGC_12731	NIPPONBARE	Japan	6	NO	6	Na	u	u	H15
IRGC_22710	NONA BOKRA	India	1	NO	1	Na	u	u	ns
IRGC_38690	NPE 253	Pakistan	6	NO	6	Na	u	u	ns
IRGC_5999	PANKHARI 203	India	5	YES	5A	Ar	P	A	H1
IRGC_8266	PEH PI NUO	China	6	NO	6	Na	u	u	ns

ID	Variety name	Country	Isozyme group	SSR genotyping	Structure group	Aroma	Genotype 8 bp del.	Genotype 3 bp ins.	Haplotype
IRGC_32571	PETA	Indonesia	1	NO	1	Na	u	u	ns
IRGC_32399	PHUDUGEY	Bhutan	5	YES	5A	Na	A	A	H4
IRGC_8948	POKKALI	Sri Lanka	1	NO	1	Na	u	u	ns
IRGC_6386	PTB 25	India	1	NO	1	Na	u	u	ns
IRGC_8952	RATHUWEE	Sri Lanka	1	NO	1	Na	u	u	ns
IRGC_8197	SHAI KUH	China	1	NO	1	Na	u	u	ns
IRGC_5418	SINTANE DIOFOR	Burkina Fasso	1	NO	m	Na	u	u	ns
IRGC_8256	SURJAMKUHI	India	2	NO	2	Na	u	u	ns
IRGC_46768	T 26	India	5	YES	m	u	A	A	H7
IRGC_105	TAICHUNG NATIVE 1	Taiwan	1	NO	1	Na	u	u	ns
IRGC_47743	TAINUNG 67	Taiwan	1	NO	1	Na	u	u	ns
IRGC_32362	TCHAMPA	India	5	YES	5A	Na	A	A	H2
IRGC_81093	TEQUING	China	1	NO	1	Na	u	u	ns
IRGC_32576	TETEP	Vietnam	1	NO	1	Na	u	u	ns
IRGC_43675	TREMBESE	Indonesia	6	NO	6	Na	u	u	ns
IRGC_33888	YELAIK MEEDON	Myanmar	5	NO	5B	u	A	A	H1

u = unknown; Ar= aromatic; NA= non-aromatic; Genotype: 8bp del. = 8 bp deletion in exon 7 in comparison with Nipponbare sequence; 3 bp ins. = 3 bp insertion in exon 13 in comparison with Nipponbare sequence; A= absent; P= present; Haplotype: ns= not sequenced

Supplementary Table 2: List of the primers used to amplify segments of the *BADH2* gene

Name	Forward primer	Reverse primer	Annealing temperature	Segment amplified	Source
**INS3**	GTCCTGTTCAATCTTGCAGC	CTTGATGCAACCATGTCATA	55°C	E13	Designed by us
**DEL8**	TGCTCCTTTGTCATCACACC	TTTCCACCAAGTTCCAGTGA	55°C	E7	Vanavichit et al. 2008
**BADH 1**	CGAAGTCCGTACCAACTGC	GGCCGTGAGCCATATACACT	55°C	5'U-I2	Kovach et al. 2009
**BADH 2**	AGTTGGAAGCATGGCTGATT	CCAGCTCAGATTTCCTCTCG	55°C	I2-E3	Kovach et al. 2009
**BADH 3**	GATTGTGGGAAGCCTCTTGA	CGATAGGCTCTTTCCGAAGAT	55°C	E3-E4	Kovach et al. 2009
**BADH 4**	ATCTTCGGAAAGAGCCTATCG	AGGAGCTACCTTCCATGTTGC	55°C	E4-E5	Kovach et al. 2009
**BADH 5**	CTATCCTCTCCTGATGGCAAC	TGGCTACTAGAATGATGCTCAAAG	55°C	E5-I6	Kovach et al. 2009
**BADH 6**	TGCTCCTTTGTCATCACACC	GGTCCAAAAGCAACCAAAGA	55°C	E7-E8	Vanavichit et al. 2008
**BADH 7**	GCCATGCCAACTGAGTAAAG	CAATTTTATTCGCTCTGTGC	55°C	E8-E9	Vanavichit et al. 2008
**BADH 8**	TGCAACATCGCGTCTTATTC	GCAACTAGCAAGAGCATACACC	55°C	E9-E10	Vanavichit et al. 2008
**BADH 9**	GCCCGTTGTTAGTGAAGGAC	GTACCATCCCCACGGCTCAT	55°C	E10-I13	Vanavichit et al. 2008
**BADH 10**	ACCTGACATCATGCCTTTGG	CCGGTCATCAGCTAACTTCC	55°C	E14 -3'U	Vanavichit et al. 2008
**BADH 11**	GCCCGTTGTTAGTGAAGGAC	CAATGGCTTCTTCTTCAGTGC	55°C	I11-E12	Designed by us
**BADH 12**	CGAGCGATGCCAGAGATTA	CGTCACTTGCTTGACGCTTA	55°C	E13-E14	Designed by us

E = exon; I = intron; U= untranslated region

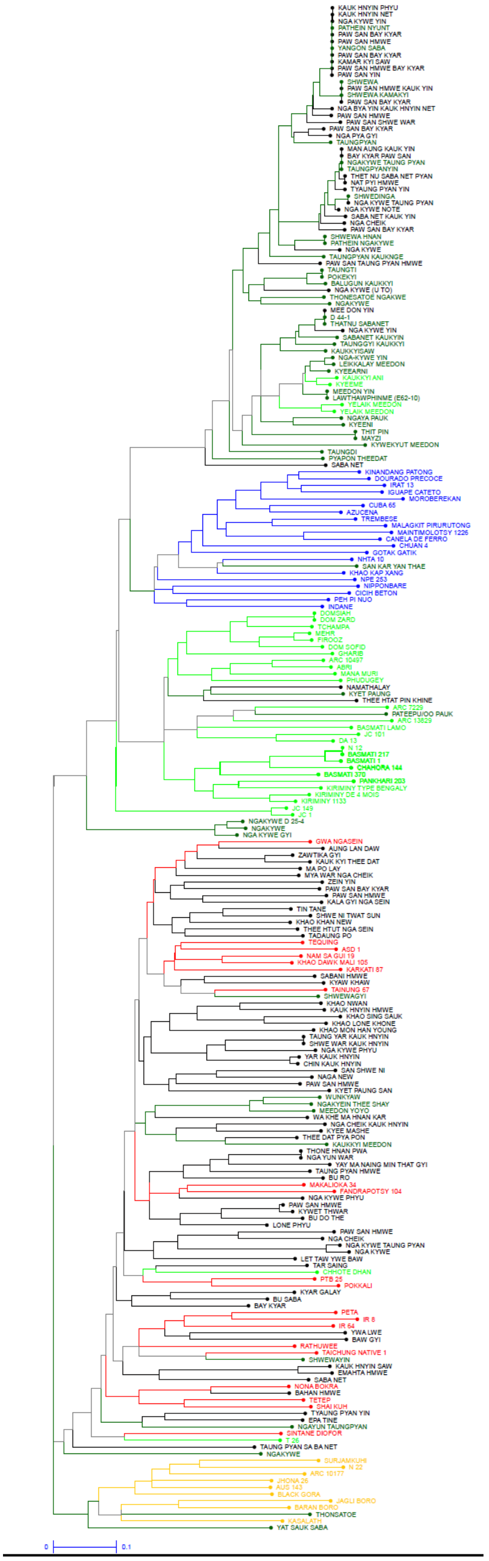

Supplementary Figure S1: NJ tree representing the relative position of Myanmar accessions and the reference set.

Colors according to enzymatic groups: 1 = indica accessions in red, 2=aus/boro in yellow, 5=sadri/basmati in light green (reference set) or dark green (Myanmar accessions based on data from Khush et al (2003)=); 6=japonica in blue; unknown group in black.

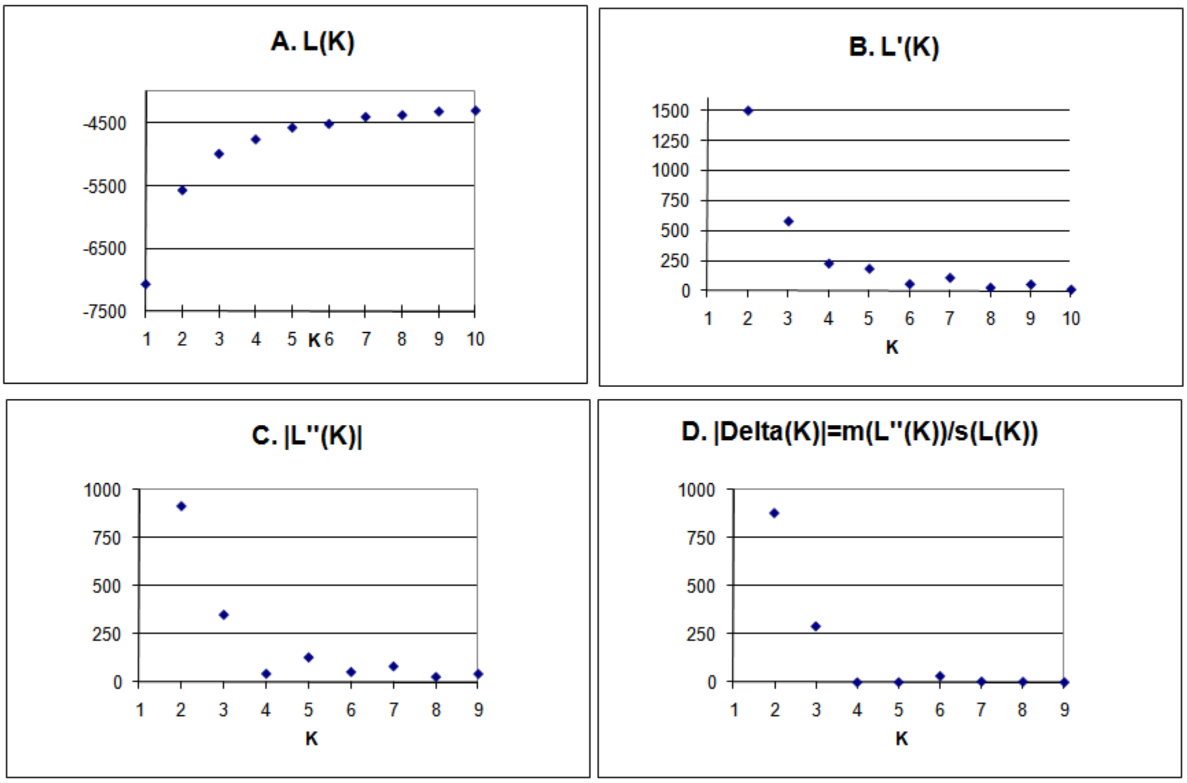

Supplementary Figure S2: Evolution of the criteria that enabled detection of the likely number of subpopulations (K) in the 227 accessions using 19 SSR markers for K values varying from 1 to 10.
A: L(k) = mean of the likelihood distribution LnP(D) over 10 runs for each K value. B: L'(K) = rate of change of the likelihood function with respect to K. C:|L''(K)| = second order rate of change of L(K) with respect to K; Delta(k) = mean |L''(K)|/standard deviation (L(K)).
