# Supplementary figures and images for: Specific patterns of genetic diversity among aromatic rice varieties in Myanmar
Source: Rice (N Y). 2012 Aug 1;5:20. doi: 10.1186/1939-8433-5-20 (PMC5520840; doi:10.1186/1939-8433-5-20)

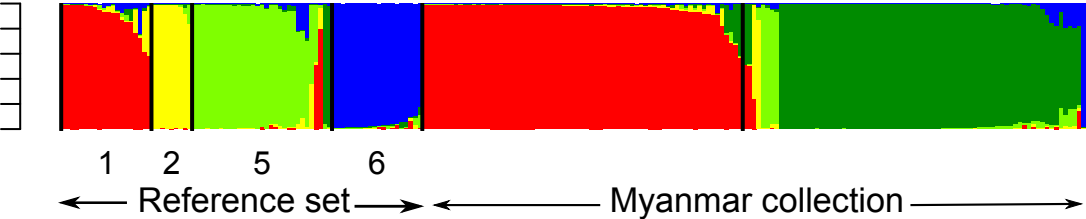

Supplement: Supplementary file 2 — Authors’ original file for figure 1 [file 12284_2011_16_MOESM2_ESM.pdf]

2.A

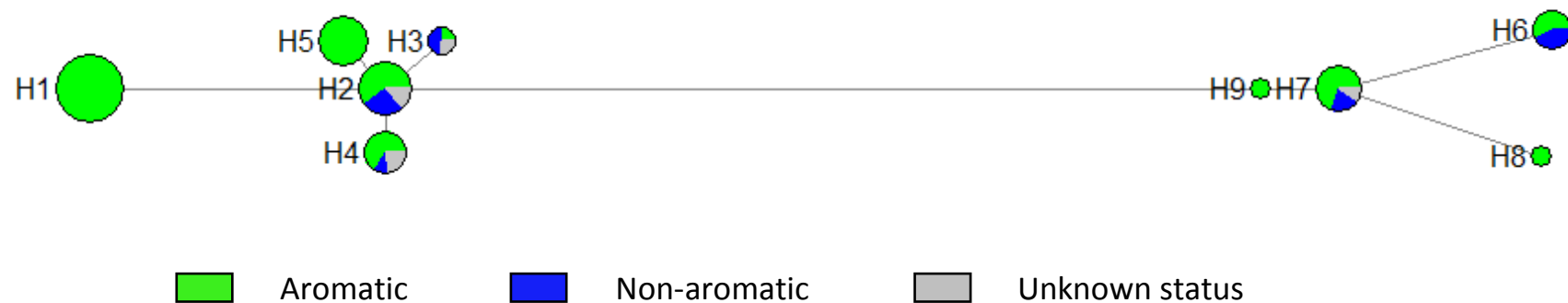

2.B

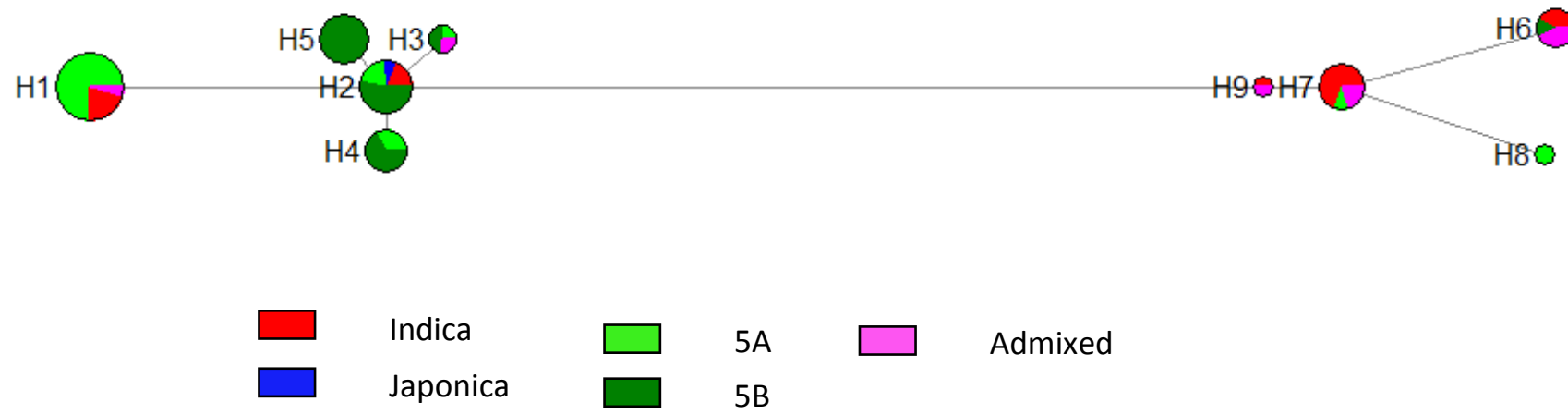

Supplement: Supplementary file 3 — Authors’ original file for figure 2 [file 12284_2011_16_MOESM3_ESM.pdf]
